# Supplementary material for: Ultrafast quantum control of ionization dynamics in krypton
Source: Nat Commun. 2018 Feb 19;9:719. doi: 10.1038/s41467-018-03122-1 (PMC5818503; doi:10.1038/s41467-018-03122-1)
Supplement: Supplementary file 1 — Supplementary Information [file 41467_2018_3122_MOESM1_ESM.pdf]

Ultrafast Quantum Control of Ionization Dynamics in Krypton

Supplementary Information

Konrad Hütten[1,2], Michael Mittermair[1,2], Sebastian O. Stock[3,4], Randolf Beerwerth[3,4], Vahe Shirvanyan[1,2], Johann Riemensberger[1,2], Andreas Duensing[1], Rupert Heider[1], Martin S. Wagner[1], Alexander Guggenmos[2], Stephan Fritzsche[3,4,5], Nikolay M. Kabachnik[6,7,8], Reinhard Kienberger[1,2] and Birgitta Bernhardt[1,5,9,†]

[1]Physics Department E11, Technical University of Munich, 85748 Garching, Germany
[2]Max Planck Institute of Quantum Optics, 85748 Garching, Germany
[3]Helmholtz-Institut Jena, 07743 Jena, Germany
[4]Theoretisch-Physikalisches Institut, Friedrich Schiller University Jena, 07745 Jena, Germany
[5]Abbe Center of Photonics, Friedrich Schiller University Jena, 07745 Jena, Germany
[6]European XFEL GmbH, 22869 Schenefeld Hamburg, Germany
[7]Skobeltsyn Institute of Nuclear Physics, Lomonosov Moscow State University, Moscow 119991, RussiaDonostia
[8]Donostia International Physics Center (DIPC), E-20018 San Sebastian/Donostia, Spain
[9]Institute of Applied Physics, Friedrich Schiller University Jena, 07745 Jena, Germany
[†]Corresponding Author: Birgitta.Bernhardt@uni-jena.de

Supplementary Note 1: Experimental details

For this experiment, we adopted and partially improved the setup used by Uiberacker et al. (for a more detailed description, see the supplementary information in M. Uiberacker et al.[1]).
A Ti:Sapphire laser system (Femtopower Compact Pro, Femtolasers GmbH) generates 25 fs, ~1.2 mJ, carrier-envelope-phase (CEP) stabilized pulses at a repetition rate of 3 kHz. These pulses are focused into a hollow core fiber filled with Neon ($P_{\mathrm{Ne}} \approx$ 1.7 bar) for spectral broadening and subsequently compressed by chirped mirrors to <5 fs (see Supplementary Figure 1), CEP-stable, ~0.5 mJ pulses with ~1.5 eV central photon energy. Injected into the evacuated beamline, the NIR laser pulses are focused into a 2 mm inner diameter neon-filled gas nozzle, generating a collinear XUV-beam via high harmonic generation (HHG).

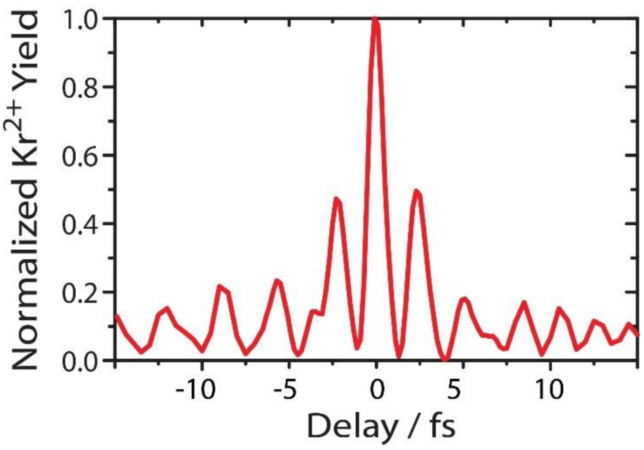

Supplementary Figure 1: Kr^{2+} autocorrelation. The trace is generated by the double mirror interferometer with NIR laser light only, witnessing near single cycle pulse duration and isolated attosecond pulse generation. This type of measurement was performed also for time zero determination before all presented ion and absorption measurements.

The stability of the XUV-flux and spectrum was significantly improved by introducing a beam pointing stabilization unit in front of the HHG-target. Isolated attosecond pulse generation was achieved by dispersion tuning for maximum amplitude and minimum duration of the Kr^{2+} autocorrelation trace generated by NIR only in the double mirror interferometer (cp. figure 1b in the main text).
For the measurements, both the NIR- and XUV-beam propagate collinearly over a distance of 3m towards the double mirror assembly. Between HHG generation and double mirror, a circular 150nm thick Zr filter is positioned in the beam center transmitting only the highly collimated XUV-beam and thus radially separating the highly collimated XUV from an outer NIR pulse, whose intensity is subsequently adjusted by an iris.
The double mirror assembly consists of an inner mirror for the XUV pulse (for its reflectivity characteristics in the XUV regime see Supplementary Figure 2) and the surrounding annulus shaped outer mirror for the NIR pulse, both sharing the same focal length (f = 125 mm) in order to focus the pulses into the interaction zone. The inner mirror is mounted on a piezo stage allowing for the introduction of a controlled delay between the NIR and XUV pulses.
The ion and absorption spectra are recorded sequentially with a reflectron type ion spectrometer by STEFAN KAESDORF - Geraete fuer Forschung und Industrie and an XUV spectrometer by McPherson Inc. having a spectral resolution of about 100 meV at 90 eV. The background pressure in the experimental chamber without krypton gas load is 7×10^{-8} mbar. For the ion measurement, the krypton gas pressure is homogenously 3.5×10^{-3} mbar in the experimental chamber while a gas nozzle is introduced to the interaction zone for the TAS measurement resulting in a gas pressure of 5×10^{-3} mbar in the chamber.

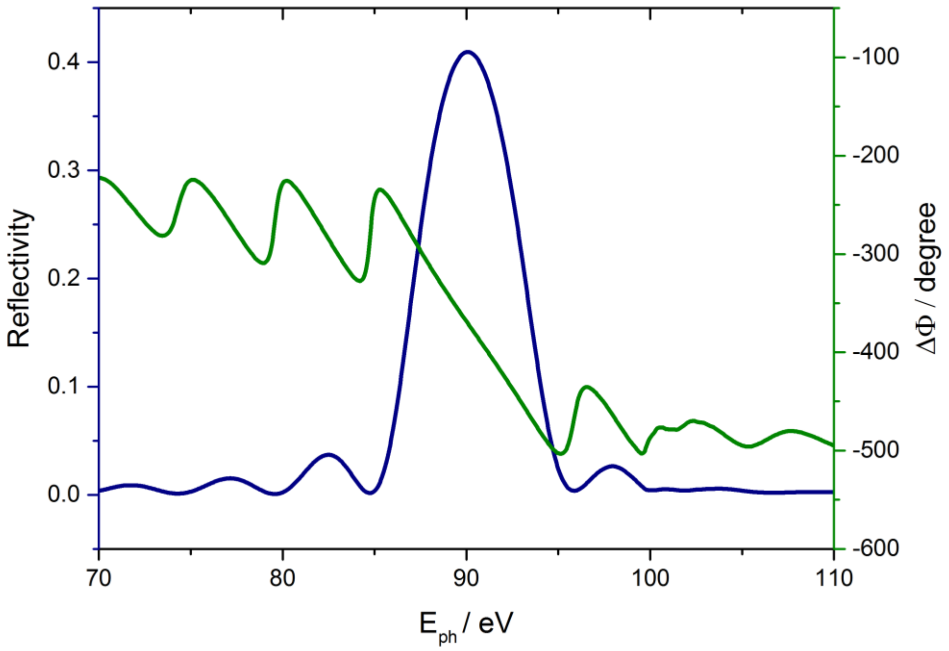

Supplementary Figure 2: XUV Reflectivity characteristics of the double-mirror. The XUV reflectivity (dark blue, left scale) is maximal at 90 eV (R_{max}=0.41), with a bandwidth of about 6 eV (FWHM). In the energy range relevant for the experiment (85 – 95 eV), the spectral phase shift $\Delta\Phi$ (green, right scale) of the reflected XUV pulse is rather linear.

Supplementary Note 2: Combined measurement of ion yield and photo absorption

Supplementary Figure 3 shows the change in the Kr absorbance and the Kr^{3+} ion yield for two different NIR intensities, (8.6 ± 1) x 10^{13} W cm^{-2}, panels a and c on the left side, and (2.9 ± 0.5) x 10^{14} W cm^{-2}, panels b and d on the right side. Panels a and b display ΔOD and the Kr^{3+} ion yield for higher delay times up to 200 fs while panels c and d provide a close-up of the pulse overlap area Δt = [-20 fs,+50 fs] (identical to figure 3 in the letter).

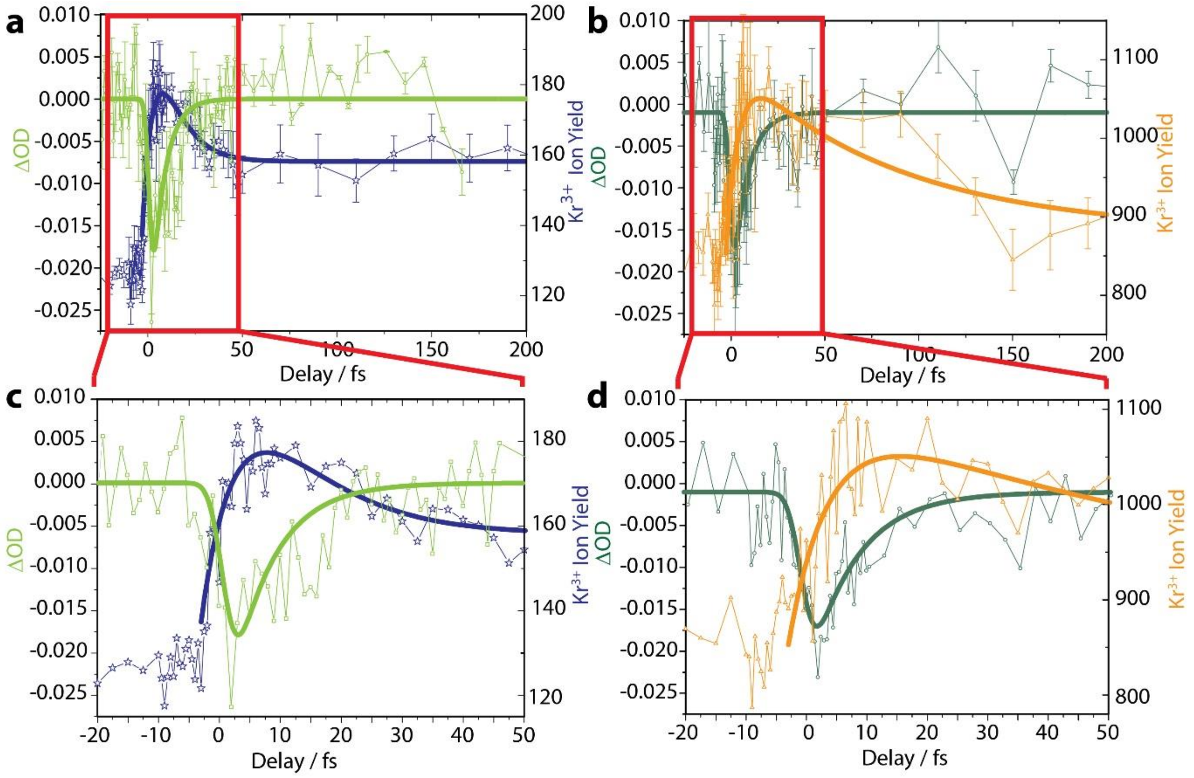

Supplementary Figure 3: Absorbance change ΔOD and Kr^{3+} ion yield vs. time delay. The change in absorbance or optical density ΔOD gives the difference of the optical density $OD = -log_{10}(I_t(\Delta t)/I_0)$ at a given time delay Δt with respect to a reference time t_R (here: t_R = -30 fs, $I_t(\Delta t)$ is the transmitted signal at time delay Δt, I_0 is the spectrum without gas). **a** At lower NIR intensities of (8.6 ± 1) x 10^{13} W cm^{-2}, the absorbance (light green, left scale) transiently drops around pulse overlap, while the Kr^{3+} ion yield (blue, right scale) shortly rises before it settles to an elevated ion yield of almost 160 counts in 20 s integration time, **b** At higher NIR intensities of (2.9 ± 0.5) x 10^{14} W cm^{-2}, the absorbance (dark green, left scale) transiently drops around pulse overlap similarly to **a**, while the Kr^{3+} ion yield (orange, right scale) shortly rises before it settles to an elevated ion yield of about 860 counts in 20 s integration time with a slower decay constant when compared to **a**. The panels **c** and **d** show close-ups of panels **a** and **b** around pulse overlap from -20 fs to +50 fs at the corresponding low and high NIR intensities, respectively. In the close-ups **c** and **d**, the error bars are not displayed for better readability.

Supplementary Note 3: NIR pre-pulse causing a transient increase of the Kr^{3+} ion yield at about 70 fs

A common feature of NIR few-cycle laser systems is the appearance of pre-/post-pulses several tens of fs before/after the few-cycle laser pulse due to inevitable oscillations in the group delay dispersion characteristics of the chirped dielectric mirrors that are used to compress the spectrally broadened

output of the laser chain. Only in the case of the highest intensity, the accompanying pulse is intense enough to influence the Kr^{3+} ion yield significantly (see figure 4c of the main text). Its presence is revealed by a xenon autocorrelation measurement (see Supplementary Figure 4a).

To account for the transient increase in the Kr^{3+} ion yield at high NIR intensities, the corresponding three elevated data points (marked in red) have been excluded from the fit, resulting in an unaffected extraction of the time constants and branching ratios.

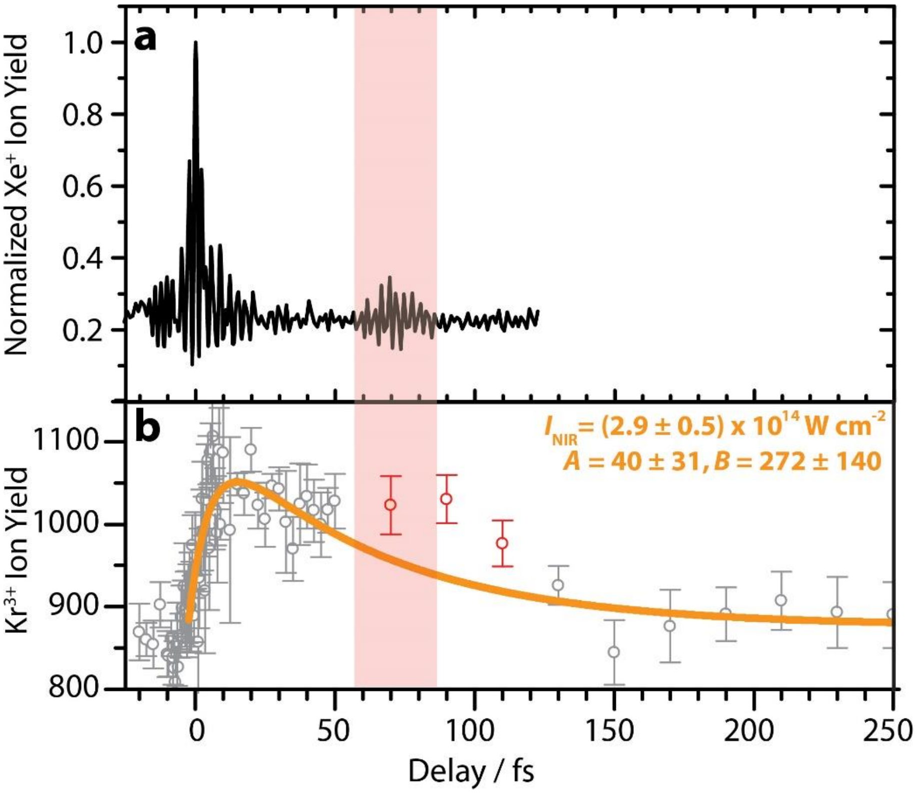

Supplementary Figure 4: Autocorrelation trace of Xe^{+} **(a)** and the Kr^{3+} ion yield change presented in the main manuscript for high NIR intensities **(b)**, both vs. time delay. The autocorrelation trace is recorded with interfering two NIR pulse replica of similar intensity in xenon, revealing a pre-pulse 70 fs before the central pulse envelope. This pre-pulse causes a transient increase of the Kr^{3+} ion yield at around 70 fs (**b**) at the NIR intensity of (2.9 ± 0.5) x 10^{14} W cm^{-2}. The affected data points (marked red) are not included into the fit. The error bars show the standard error of the average of six measurements.

Supplementary Note 4: Qualitative analysis of the time dependence of the Kr^{3+} ion yield for different NIR intensities

Here we present a qualitative analysis of the results for the Kr^{3+} ion yield, shown in Fig. 4, paying particular attention to the dependence of the results on the NIR intensity. For this analysis, it is convenient to use a more detailed scheme of the Auger transitions as presented in Fig. 1. For the more detailed energy scheme, please see figure 1 in A. Verhoef et al.[2].

Direct and indirect ionization

By comparing the XUV radiation generated in the experiment with photon-energy resolved measurements[3] of the ion yield in Kr, the rate of direct ionization with respect to resonant excitation can be estimated. The comparison yields about 16.5 % of direct ionization. The majority of the XUV photons consequently contribute to the resonant excitation of the $3d^{-1}{}_{5/2}np$ and $3d^{-1}{}_{3/2}np$ states. The

directly produced Kr^{+} ions ($3d^{-1}$) decay via Auger transitions with a decay constant 7.5 fs [4] mainly to the states of the Kr^{2+} ions lying below the threshold of direct Kr^{3+} production. However, about 30% of the directly ionized $3d^{-1}$ decay to Kr^{3+} [5]. This yield of Kr^{3+} ions is independent of the presence of the NIR pulse and hence, does neither depend on the NIR intensity nor the time delay and may be considered as a permanent background. The states of Kr^{2+} that are emerging from the decay of the $3d^{-1}$ vacancy and that are lying below the Kr^{3+} threshold, i.e. the states with the configurations $4s^{-2}$, $4p^{-4}\,4d^{2}$, $4p^{-3}4d$, and $4s^{-1}4p^{-1}$ can be further excited by the NIR laser field to Kr^{3+}. This leads to the increase of the Kr^{3+} yield within the first ~10 fs after excitation. This increase is more significant for larger NIR intensities. However, for large positive time delays (> 70 fs, XUV pulse arrives well before NIR pulse), this mechanism does not contribute.

Negative time delays

At negative time delays (NIR pulse comes first), the NIR field can excite or ionize the neutral Kr atoms. The following XUV pulse can further ionize the inner 3*d* shell of the Kr^{+} ion resulting in a highly excited state of Kr^{2+} which decays to Kr^{3+} (not shown in the energy diagram of Verhoef et al.[2]). Our experiment shows that at negative time delays, the Kr^{3+} yield increases with the NIR intensity, which confirms that at intensities used in this work, the ionization of Kr atoms is not saturated [6].

Large positive time delays

At very large positive time delays (> 200 fs), practically all Auger decays are already proceeded. Only those excited ionic states survive which can further decay only by radiative decays which have much longer decay constants of several picoseconds and more. They are practically stable on the considered time scale. These are the states of Kr^{2+} ions lying below Kr^{3+} threshold. They can be, however, further ionized by the delayed NIR pulse increasing the yield of Kr^{3+} ions. In fact, we observe such an increase with increasing NIR intensity at large time delays.

Small positive time delays

The most interesting part of the Kr^{3+} yield as a function of time delay is the Kr^{3+} behavior between Δt = 0 fs and 50 fs. The initial increase of that yield at small positive delays is mainly determined by the XUV excitation and Auger decay (first step) of the $3d^{-1}np$ resonances which can directly decay to the Kr^{3+} ions [7] or populate the intermediate states of Kr^{+} (groups of states called *A* and *B* in Fig 1a) which can be further ionized by the NIR laser [6]. An additional contribution is due to the direct XUV ionization and decay of the $3d^{-1}$ states (see above). Besides, the population of the $3d^{-1}$ vacancy states can be increased if the Rydberg electron *np* is removed by the NIR field before the resonance has decayed, which leads to further increase of the Kr^{3+} yield at small positive delays. Since the decay constants of all resonant $3d^{-1}np$ states[7] and of the 3*d* vacancy[5] are close to each other and equal to about 7.9 fs, the increase of the Kr^{3+} yield at small delays is characterized by this constant.

The time development of the Kr^{3+} ion yield at larger time delays (Δt = [10 fs,50 fs]) is determined by two competing processes: the second step Auger decay of the intermediate Kr^{+} states to the states of Kr^{2+} and double ionization of those intermediate states by the NIR pulse to the states of Kr^{3+}. Thus, by changing the time delay between the XUV and NIR pulses, one can probe the decay constant of the second step in the Auger cascades. When the IR intensity increases, different groups of the Kr^{+} state contribute which have different time constants. This phenomenon was used in the present work for disentangling the decay paths of the Auger cascades.

Supplementary Note 5: Theoretical calculations for the decay of the Kr $3d^{-1}np$ states

In order to estimate the effective lifetimes of the observed levels in Kr^+, we study the decay cascade that follows the resonant excitation of the Kr $3d^{-1}np$ states by the XUV beam. We perform fully relativistic atomic structure calculations based on the multiconfiguration Dirac-Fock (MCDF) method[8]. The wave functions were generated using the GRASP package[9]. Based on these wave functions, the Einstein coefficients for the resonant excitation and the rates of the subsequent Auger decay processes were calculated using the programs of the RATIP package[10].

Similarly to our previous work on oxygen anions[11] and neutral neon[12], we utilize the computed transition rates to construct the decay tree that includes all major de-excitation pathways due to sequential Auger decays. Shake transitions of the excited 5*p* or 6*p* spectator electron play a crucial role and are included to the *np* subshells with n = 5, 6, 7. Direct double Auger processes are not considered here. Based on the relative population of the fine-structure levels of Kr^+, the effective lifetimes are calculated as a weighted average of the lifetimes of the individual levels.

For the initial excitation by the XUV beam, we considered the population of the $3d^{-1}5p$ and $3d^{-1}6p$ levels, taking into account the spectral line shape of the beam (cp. Figure 1c). The lifetimes of the populated $3d^{-1}np$ levels are around 7.1–7.2 fs based on our calculations, in good agreement with the literature [13]. The excited levels decay via a two-step Auger cascade (see Supplementary Figure 5).

First step

The first step of the cascade is dominated by spectator Auger decays to several configurations of Kr^+, participator processes are negligible according to our calculations. The branching fractions for the decays to different configurations in Kr^+ are listed in Supplementary Table 1 (cp. also Supplementary Figure 5).

Configuration	Branching
$4p^{-2}\,np$	32 %
$4s^{-1}4p^{-1}\,np/4p^{-3}4d\,np$	57 %
$4s^{-2}\,np$	11 %

Supplementary Table 1: Branching ratios for the decays to different Kr^+ configurations.

Here, we do not differentiate between the $4s^{-1}4p^{-1}\,np$ and $4p^{-3}4d\,np$ configurations, since they strongly mix into each other and cannot be clearly distinguished. The same applies to the $4s^{-1}4p^{-1}$ and $4p^{-3}4d$ configurations of Kr^{2+}.

No.	Energy / eV	Lifetime / fs	No.	Energy / eV	Lifetime / fs
1	46.3	48	7	60.1	44
2	50.0	84	8	60.8	10
3	51.4	370	9	62.3	19
4	55.2	7	10	68.5	3
5	57.2	4	11	72.0	7
6	58.7	20	12	73.4	16

Supplementary Table 2: Lifetimes of selected Kr^+ levels. The numbers in the first column refer to the numbers above the spectra in Supplementary Figure 5. Energies are given relative to the ground level of neutral krypton.

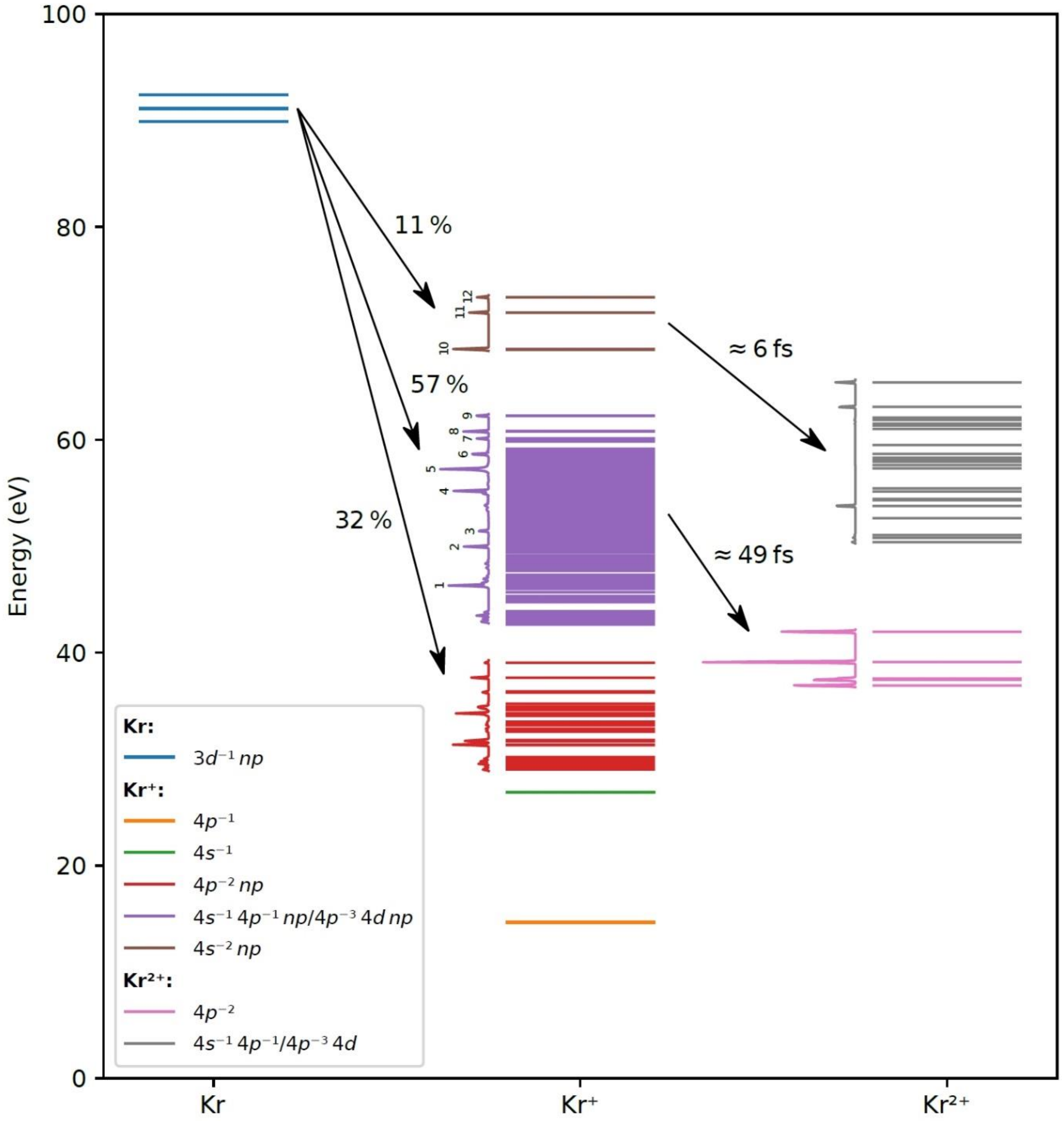

Supplementary Figure 5: Auger transitions after resonant excitation of the Kr $3d^{-1}$ *np* states. All (calculated) energies are given relative to the ground level of neutral krypton. The vertical spectra next to the level structure show the relative population of each level. The numbers above the peaks in the spectra for Kr^+ refer to the first column of Supplementary Table 2, which shows the lifetimes of these levels.

Second step

In the second step, the $4s^{-2}np$ states decay mainly to three levels of the $4s^{-1}4p^{-1}/4p^{-3}4d$ configurations of Kr^{2+}, while the $4s^{-1}4p^{-1}np/4p^{-3}4dnp$ levels decay to the $4p^{-2}$ ground configuration of Kr^{2+}.
The lifetimes of the populated Kr^+ levels due to these transitions range from a few femtoseconds to several hundred femtoseconds. To illustrate this, Supplementary Table 2 lists the lifetimes of Kr^+ levels which are dominantly populated during the first step of the cascade. The effective lifetimes of these configurations are 6 fs for the $4s^{-2}np$ levels and 49 fs for the $4s^{-1}4p^{-1}np/4p^{-3}4dnp$ levels. However, we believe these lifetimes to be only a rough estimate due to the following:

We did not include the so called multiplet-changing transitions $4p^{-2}np \rightarrow 4p^{-2}$ and $4s^{-1}4p^{-1}np$ /$4p^{-3}4dnp$ $\rightarrow$ $4s^{-1}4p^{-1}$/$4p^{-3}4d$ in our calculations (even though some appear energetically possible), because according to our experience their rates are highly sensitive to correlation effects and are often misestimated in such a calculation. Nevertheless, in reality these transitions would likely contribute to the decay of the $4s^{-1}4p^{-1}np$/$4p^{-3}4dnp$ levels, leading to a shorter lifetime. While multiplet-changing transitions play a dominant role in the decay cascades of resonantly excited neon[12], test calculations performed by us suggest that, in the case of the krypton $4s^{-1}4p^{-1}np$ /$4p^{-3}4dnp$ levels, their contributions to the total lifetimes are likely not as large and we estimate the error that is due to not including these transitions to be less than 20%.The first step of the cascade is heavily influenced by shake-ups of the 5*p*/6*p* spectator electron to higher *np* subshells. For this purpose, we included shake processes to the 5*p*, 6*p*, and 7*p* subshells in the computations. However, the analysis shows that shake-ups to even higher subshells likely occur, especially for an initial 6p electron, and are not entirely negligible. These shake-ups influence the effective lifetimes, since the lifetime increases for higher subshells. This becomes evident when looking at the lifetimes of the $4p^6np$ levels that are listed in Supplementary Table 3. Considering shake processes to even higher subshells (at least 8*p* and 9*p*) should therefore lead to a slight increase of the effective lifetimes.

**Configuration**	**Lifetime / fs**
$4s^{-2}5p$	3
$4s^{-2}6p$	7
$4s^{-2}7p$	16

Supplementary Table 3: Lifetimes of selected $4p^6np$ levels.

Supplementary References:

1. Uiberacker, M. *et al.* Attosecond real-time observation of electron tunnelling in atoms. *Nature* **446,** 627–632 (2007).
2. Verhoef, A. J. *et al.* Time-and-energy-resolved measurement of Auger cascades following Kr 3d excitation by attosecond pulses. *New J. Phys.* **13,** 113003 (2011).
3. Lablanquie, P. *et al.* Photoemission of threshold electrons in the vicinity of the xenon 4d hole: dynamics of Auger decay. *J. Phys. B At. Mol. Opt. Phys.* **35,** 3265 (2002).
4. Jurvansuu, M., Kivimäki, A. & Aksela, S. Inherent lifetime widths of Ar $2p^{-1}$, Kr $3d^{-1}$, Xe $3d^{-1}$, and Xe $4d^{-1}$ states. *Phys. Rev. A* **64,** 12502 (2001).
5. Brünken, S. *et al.* Decay of the Ar $2s^{-1}$ and $2p^{-1}$ and Kr $3p^{-1}$ and $3d^{-1}$ hole states studied by photoelectron-ion coincidence spectroscopy. *Phys. Rev. A* **65,** 42708 (2002).
6. Uphues, T. *et al.* Ion-charge-state chronoscopy of cascaded atomic Auger decay. *New J. Phys.* **10,** 25009 (2008).
7. Lablanquie, P. & Morin, P. Double ionization following the $3d_{5/2}$ to 5p excitation in Kr. *J. Phys. B At. Mol. Opt. Phys.* **24,** 4349–4362 (1991).
8. Grant, I. P. *Relativistic Quantum Theory of Atoms and Molecules*. (Springer New York, 2007). doi:10.1007/978-0-387-35069-1
9. Jönsson, P., He, X., Froese Fischer, C. & Grant, I. P. The grasp2K relativistic atomic structure package. *Comput. Phys. Commun.* **177,** 597 (2007).
10. Fritzsche, S. The Ratip program for relativistic calculations of atomic transition, ionization and recombination properties. *Comput. Phys. Commun.* **183,** 1525 (2012).
11. Schippers, S. *et al.* Prominent role of multielectron processes in K -shell double and triple photodetachment of oxygen anions. *Phys. Rev. A* **94,** 41401 (2016).
12. Stock, S., Beerwerth, R. & Fritzsche, S. Auger cascades in resonantly excited neon. *Phys. Rev. A* **95,** 53407 (2017).
13. Sairanen, O.-P., Kivimäki, A., Nõmmiste, E., Aksela, H. & Aksela, S. High-resolution pre-edge structure in the inner-shell ionization threshold region of rare gases Xe, Kr, and Ar. *Phys. Rev. A* **54,** 2834–2839 (1996).
